# Medical Home-Truths.—II. Medicine in General
*The previous article appeared on January 13, p. 304.


**Published:** 1917-01-20

**Authors:** 


### MEDICAL HOME-TRUTHS.

### II. Medicine in General.*

In The Hospital for August 22, 1908, this writer had it: "Every medical man who has taken the trouble to examine the sources of the statements in his text-books will be in full sympathy with the cosmopolitanism of science; and in proportion to the extent of such investigation he will be filled with respect for the patient, sagacious labours of the German, who latterly has so outworked those of other nationalities in medical science. So far as outside knowledge warrants the expression of an opinion, it seems to be the same with many other branches of learning. Nearly everywhere the appeal of the critic is to some *Zeitschrift*, or to ponderous volumes (jocularly called handbooks) published in Leipzig or Berlin. The advance of the Western nations, again, is dependent upon scientific research in its widest sense, and therefore upon the teachings of an intellectual oligarchy, to which the German people has shown itself much more heedful than, for instance, our own nation. Altogether it seems likely that if the Teuton should choose to become an active rival, he must prove an exceedingly formidable one."

This is but an humble variation on the theme of Mr. Frederic Harrison, whose advice was to fraternise with the *savant*, the poet, the artist of the Fatherland; but to keep powder dry: and before him, on that of Meredith, in "One of our Conquerors"; and after him, on passages in Mr. Wells's masterpiece "The New Machiavelli," so much truer and more profound than his war-time effusions. Since 1908 that theme has received an unexampled popular demonstration. The man in the street knows now what the student did then. Yet there are many medical men, even in somewhat leading positions, who would doubt its holding good in the province of medicine. The ultimate reason of this is that the British are poor linguists. It is not perceived that a few months' visit to Vienna will not make up for subsequent life-long failure to keep in touch with Continental medicine. That surgeon to the hospital of a large industrial city, that young public-health man fresh from beyond Tweed, so secure in the mutual good opinion of his fellow-graduates—it is hard for such as these to realise that *confrères* in little Norwegian or Swiss towns are their masters, simply because the latter belong to a huge Continental school. There is a certain well-known provincial university, no single member of the medical faculty of which reads German. The hospital serves a population larger than that of Bordeaux, far larger than that of Freiburg; but what a vast difference in the calibre and prestige of the respective staffs, and in the respective standards of achievement! One often sees the "Lyons teaching" on certain points quoted in current medical literature; but does one ever see so quoted the teaching of any provincial city in England, large as many of them are? In 1877 Sir William Jenner wrote warning a consumptive against "entrusting his vile body to the Davos doctors." But now, indeed any time for the last ten years, all previous classifications of pulmonary tuberculosis have been ousted by that of Turban.

In fine, so it comes that, in this present time of stress, by far the best approved substitutes for the German invented drug salvarsan are the French compounds hectin and galyl, a fact in all its bearings deeply discreditable to British medicine. It is not denied that, if Ehrlich be left out of count, Sir Almroth Wright and the late Sir Victor Horsley were a pair of medical investigators equal to any other in Europe, while the same may be said of Worth and Heath as clinical specialists. Sir Ronald Ross, too, is a Nobel prizeman. It is, however, curious that the home recognition of these five has been less than that accorded to medical men to whom they stand, to use a modern figure, in the relation of aeroplane to taxi-cab.

But it is not our purpose to recommend here remedies for the state of matters we have endeavoured faithfully to describe, other than to say that no good will be done by depreciatory references to Austro-German medicine introduced into scientific articles, spoiling the pure objectivity necessary to such work. This is an error into which the other side does not fall nearly so frequently. A writer on food values and the feeding question makes some obviously extravagant claims, after the manner of the writer of a leading article. He is rebuked for allowing patriotism to colour his science. The reviewer of a brochure on the psychological traits of the English says little beyond intimating that the good points of the English character are fully treated. What has given us our present subject is the curious lack of information thereon, or the curious face-saving treatment, which is to be seen so often in circles both medical and lay. These two phenomena are equally repellent to the truth-lover, of whom there should be many in a scientific profession, particularly that of medicine. For surely in medicine the Socratic saying that knowledge is virtue is justified, if anywhere; so that one might add the corollary that ignorance is criminal. If, then, the oxymoron now concluded has contributed something at once true, topical, and important, its initial bitter flavour may be forgiven.

* The previous article appeared on January 13, p. 304.

